# 2-Amino-4-methyl­pyridinium 2-hy­droxy­benzoate

**DOI:** 10.1107/S160053681002965X

**Published:** 2010-07-31

**Authors:** Madhukar Hemamalini, Hoong-Kun Fun

**Affiliations:** a X-ray Crystallography Unit, School of Physics, Universiti Sains Malaysia, 11800 USM, Penang, Malaysia

## Abstract

The asymmetric unit of the title mol­ecular salt, C_6_H_9_N_2_^+^·C_7_H_5_O_3_^−^, contains two cations and two anions. Both the salicylate anions contain an intra­molecular O—H⋯O hydrogen bond, which generates an *S*(6) ring. Both the 2-amino-4-methyl­pyridine mol­ecules are protonated at their pyridine N atoms. In the crystal, both cations form two N—H⋯O hydrogen bonds to their adjacent anions, forming ion pairs. Further N—H⋯O links generate sheets lying parallel to the *ab* plane. In addition, weak C—H⋯O bonds and aromatic π–π stacking inter­actions [centroid–centroid distances = 3.5691 (9) and 3.6215 (9) Å] are observed between the cations and anions.

## Related literature

For related structures, see: Navarro Ranninger *et al.* (1985 ▶); Luque *et al.* (1997 ▶); Qin *et al.* (1999 ▶); Jin *et al.* (2001); Albrecht *et al.* (2003 ▶); Kvick & Noordik (1977 ▶). For hydrogen-bond motifs, see: Bernstein *et al.* (1995 ▶). For bond-length data, see: Allen *et al.* (1987 ▶). For the stability of the temperature controller used in the data collection, see: Cosier & Glazer (1986 ▶).
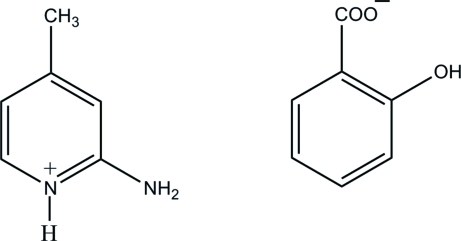


## Experimental

### 

#### Crystal data

C_6_H_9_N_2_^+^·C_7_H_5_O_3_^−^
*M* *_r_* = 246.26
Triclinic, 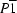

*a* = 7.2417 (2) Å
*b* = 12.5520 (3) Å
*c* = 14.7699 (3) Å
α = 68.752 (2)°
β = 82.038 (2)°
γ = 88.824 (2)°
*V* = 1238.58 (5) Å^3^
*Z* = 4
Mo *K*α radiation
μ = 0.10 mm^−1^
*T* = 100 K
0.32 × 0.10 × 0.04 mm

#### Data collection

Bruker APEXII CCD diffractometer
Absorption correction: multi-scan (*SADABS*; Bruker, 2009 ▶) *T*_min_ = 0.971, *T*_max_ = 0.996
23090 measured reflections
8280 independent reflections
5112 reflections with *I* > 2σ(*I*)
*R*_int_ = 0.047

#### Refinement

*R*[*F* ^2^ > 2σ(*F* ^2^)] = 0.056
*wR*(*F* ^2^) = 0.155
*S* = 1.00
8280 reflections
359 parameters
H atoms treated by a mixture of independent and constrained refinement
Δρ_max_ = 0.38 e Å^−3^
Δρ_min_ = −0.28 e Å^−3^

### 

Data collection: *APEX2* (Bruker, 2009 ▶); cell refinement: *SAINT* (Bruker, 2009 ▶); data reduction: *SAINT*; program(s) used to solve structure: *SHELXTL* (Sheldrick, 2008 ▶); program(s) used to refine structure: *SHELXTL*; molecular graphics: *SHELXTL*; software used to prepare material for publication: *SHELXTL* and *PLATON* (Spek, 2009 ▶).

## Supplementary Material

Crystal structure: contains datablocks global, I. DOI: 10.1107/S160053681002965X/hb5564sup1.cif

Structure factors: contains datablocks I. DOI: 10.1107/S160053681002965X/hb5564Isup2.hkl

Additional supplementary materials:  crystallographic information; 3D view; checkCIF report

## Figures and Tables

**Table 1:** Hydrogen-bond geometry (Å, °)

*D*—H⋯*A*	*D*—H	H⋯*A*	*D*⋯*A*	*D*—H⋯*A*
O3*A*—H1*A*3⋯O2*A*	0.99 (2)	1.61 (2)	2.5310 (16)	154 (2)
N1*A*—H1*NA*⋯O1*B*^i^	0.99 (2)	1.71 (2)	2.6965 (17)	174 (2)
N2*A*—H2*NA*⋯O1*A*^ii^	0.90 (2)	1.99 (2)	2.8645 (19)	164 (2)
O3*B*—H1*B*3⋯O2*B*	0.94 (3)	1.62 (3)	2.5179 (16)	158 (2)
N2*A*—H3*NA*⋯O2*B*^i^	0.94 (2)	1.91 (2)	2.8468 (18)	178 (2)
N1*B*—H1*NB*⋯O2*A*	0.96 (2)	1.76 (2)	2.7186 (17)	172.7 (17)
N2*B*—H2*NB*⋯O1*A*	0.96 (2)	1.84 (2)	2.7976 (18)	177.0 (16)
N2*B*—H3*NB*⋯O1*B*^iii^	0.93 (2)	1.88 (2)	2.8097 (19)	174.3 (13)
C8*B*—H8*BA*⋯O2*B*^iv^	0.93	2.47	3.357 (2)	159
C10*B*—H10*B*⋯O3*B*	0.93	2.38	3.039 (2)	128

Symmetry codes: (i) 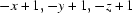; (ii) 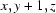; (iii) 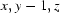; (iv) 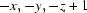.

**Fig. 1.**
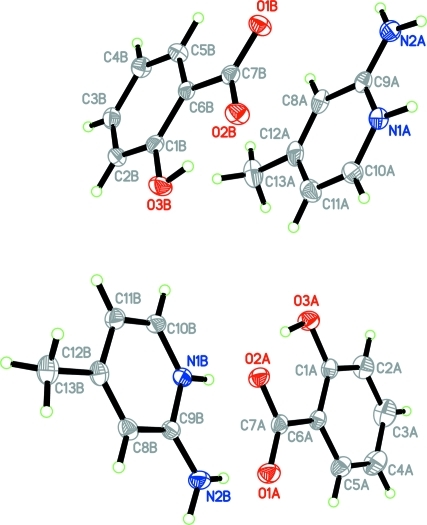
The asymmetric unit of the title compound. Displacement ellipsoids are drawn at the 50% probability level.

**Fig. 2.**
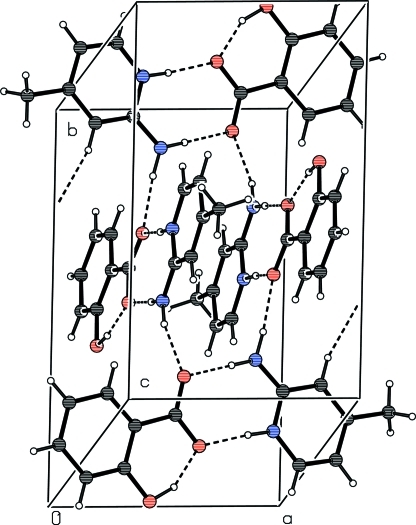
Hydrogen bonding patterns in compound (I).

## supplementary crystallographic information

### Comment

There are numerous examples of 2-amino-substituted pyridine compounds in which the 2-aminopyridines act as neutral ligands (Navarro Ranninger *et al.*, 1985; Luque *et al.*, 1997; Qin *et al.*, 1999) or as protonated cations (Luque *et al.*, 1997; Jin *et al.*, 2001; Albrecht *et al.*, 2003). In order to study some hydrogen bonding interactions, the synthesis and structure of the title salt, (I), is presented here.

The asymmetric unit of the title compound consists of two crystallographically independent 2-amino-4-methylpyridinium cations (A and B) and two salicylate anions (A and B) (Fig. 1). Each 2-amino-4-methylpyridinium cation is planar, with a maximum deviation of 0.004 (1) Å for atom N1A in cation A and 0.006 (2) Å for atom C11B in cation B. In the cations, protonation at atoms N1A and N1B lead to a slight increase in the C9A—N1A—C10A [122.06 (14)°] and C9B—N1B—C10B [121.76 (13)°] angles compared to those observed in an unprotonated structure (Kvick & Noordik, 1977). The bond lengths (Allen *et al.*, 1987) and angles are normal.

In the crystal structure (Fig. 2), the carboxylate groups of each salicylate anions interact with the corresponding 2-amino-4-methylpyridinium cations via a pair of N—H···O hydrogen bonds forming an *R*_2_^2^(8) ring motif (Bernstein *et al.*, 1995).  Furthermore, these motifs are connected via N—H···O hydrogen bonds, forming a two-dimensional network parallel to the *ab*-plane.  There is an intramolecular O—H···O hydrogen bond in the salicylate anions, which generates an *S*(6) ring motif. In addition, weak C—H···O and π–π interactions are observed between the cation-anion pairs, [Cg1(N1A/C8A–C12A)& Cg4(C1A–C6A)] and [Cg2(N1B/C8B–C12B) & Cg3(C1B–C6B)], with centroid-centroid distances of 3.5691 (9) Å (1+x, y, z) and 3.6215 (9) Å (-1+x, y, z), respectively.

### Experimental

A hot methanol solution (20 ml) of 2-amino-4-methylpyridine (54 mg, Aldrich) and salicylic acid (69 mg, Merck) were mixed and warmed over a heating magnetic stirrer hotplate for a few minutes. The resulting solution was allowed to cool slowly at room temperature and colourless needles of (I) appeared after a few days.

### Refinement

Atoms H1A3, H1B3,H1NA, H2NA, H3NA, H1NB, H2NB, H3NB were located from a difference Fourier map and were refined freely [N–H= 0.90 (2)– 0.99(20 Å and O–H =0.94 (2)–0.99 (2) Å]. The remaining hydrogen atoms were positioned geometrically [C–H = 0.93 or 0.96 Å] and were refined using a riding model, with *U*_iso_(H) = 1.2 or 1.5 *U*_eq_(C).  A rotating group model was used for the methyl group.

### Crystal data

**Table:**

C_6_H_9_N_2_^+^·C_7_H_5_O_3_^−^	*Z* = 4
*M**_r_* = 246.26	*F*(000) = 520
Triclinic, *P*1	*D*_x_ = 1.321 Mg m^−^^3^
Hall symbol: -P 1	Mo *K*α radiation, λ = 0.71073 Å
*a* = 7.2417 (2) Å	Cell parameters from 3981 reflections
*b* = 12.5520 (3) Å	θ = 2.7–31.4°
*c* = 14.7699 (3) Å	µ = 0.10 mm^−^^1^
α = 68.752 (2)°	*T* = 100 K
β = 82.038 (2)°	Needle, colourless
γ = 88.824 (2)°	0.32 × 0.10 × 0.04 mm
*V* = 1238.58 (5) Å^3^	

### Data collection

**Table:**

Bruker APEXII CCD diffractometer	8280 independent reflections
Radiation source: fine-focus sealed tube	5112 reflections with *I* > 2σ(*I*)
graphite	*R*_int_ = 0.047
φ and ω scans	θ_max_ = 31.6°, θ_min_ = 1.5°
Absorption correction: multi-scan (*SADABS*; Bruker, 2009)	*h* = −10→10
*T*_min_ = 0.971, *T*_max_ = 0.996	*k* = −15→18
23090 measured reflections	*l* = −21→21

### Refinement

**Table:**

Refinement on *F*^2^	Primary atom site location: structure-invariant direct methods
Least-squares matrix: full	Secondary atom site location: difference Fourier map
*R*[*F*^2^ > 2σ(*F*^2^)] = 0.056	Hydrogen site location: inferred from neighbouring sites
*wR*(*F*^2^) = 0.155	H atoms treated by a mixture of independent and constrained refinement
*S* = 1.00	*w* = 1/[σ^2^(*F*_o_^2^) + (0.0755*P*)^2^] where *P* = (*F*_o_^2^ + 2*F*_c_^2^)/3
8280 reflections	(Δ/σ)_max_ < 0.001
359 parameters	Δρ_max_ = 0.38 e Å^−^^3^
0 restraints	Δρ_min_ = −0.28 e Å^−^^3^

### Special details

**Table:**

Experimental. The crystal was placed in the cold stream of an Oxford Cryosystems Cobra open-flow nitrogen cryostat (Cosier & Glazer, 1986) operating at 100.0 (1) K.
Geometry. All s.u.'s (except the s.u. in the dihedral angle between two l.s. planes) are estimated using the full covariance matrix. The cell s.u.'s are taken into account individually in the estimation of s.u.'s in distances, angles and torsion angles; correlations between s.u.'s in cell parameters are only used when they are defined by crystal symmetry. An approximate (isotropic) treatment of cell s.u.'s is used for estimating s.u.'s involving l.s. planes.
Refinement. Refinement of F^2^ against ALL reflections. The weighted R-factor wR and goodness of fit S are based on F^2^, conventional R-factors R are based on F, with F set to zero for negative F^2^. The threshold expression of F^2^ > 2σ(F^2^) is used only for calculating R-factors(gt) etc. and is not relevant to the choice of reflections for refinement. R-factors based on F^2^ are statistically about twice as large as those based on F, and R- factors based on ALL data will be even larger.

### Fractional atomic coordinates and isotropic or equivalent isotropic displacement parameters (Å^2^)

**Table:**

	*x*	*y*	*z*	*U*_iso_*/*U*_eq_	
O1A	0.68689 (15)	−0.11656 (9)	0.22307 (8)	0.0266 (3)	
O2A	0.61939 (14)	0.06247 (9)	0.20906 (8)	0.0243 (2)	
O3A	0.85815 (16)	0.22440 (9)	0.12227 (8)	0.0255 (2)	
C1A	0.9761 (2)	0.14446 (13)	0.10815 (11)	0.0204 (3)	
C2A	1.1562 (2)	0.18050 (15)	0.05960 (12)	0.0290 (4)	
H2AA	1.1918	0.2575	0.0369	0.035*	
C3A	1.2811 (2)	0.10169 (17)	0.04530 (13)	0.0366 (4)	
H3AA	1.4012	0.1260	0.0133	0.044*	
C4A	1.2299 (2)	−0.01322 (16)	0.07799 (13)	0.0350 (4)	
H4AA	1.3150	−0.0660	0.0684	0.042*	
C5A	1.0506 (2)	−0.04874 (14)	0.12505 (12)	0.0263 (3)	
H5AA	1.0156	−0.1257	0.1462	0.032*	
C6A	0.9217 (2)	0.02859 (13)	0.14131 (10)	0.0198 (3)	
C7A	0.7306 (2)	−0.01258 (12)	0.19453 (11)	0.0200 (3)	
N1A	0.68931 (17)	0.46245 (11)	0.40308 (10)	0.0214 (3)	
N2A	0.74130 (19)	0.65677 (12)	0.35534 (11)	0.0247 (3)	
C8A	0.6621 (2)	0.57734 (14)	0.23833 (11)	0.0239 (3)	
H8AA	0.6670	0.6489	0.1885	0.029*	
C9A	0.6984 (2)	0.56827 (13)	0.33225 (11)	0.0207 (3)	
C10A	0.6458 (2)	0.36749 (13)	0.38600 (12)	0.0243 (3)	
H10A	0.6395	0.2966	0.4368	0.029*	
C11A	0.6114 (2)	0.37414 (15)	0.29629 (13)	0.0281 (4)	
H11A	0.5826	0.3084	0.2852	0.034*	
C12A	0.6199 (2)	0.48242 (15)	0.21964 (12)	0.0257 (3)	
C13A	0.5855 (2)	0.49005 (17)	0.11939 (13)	0.0338 (4)	
H13A	0.6056	0.5677	0.0745	0.051*	
H13B	0.4593	0.4655	0.1225	0.051*	
H13C	0.6698	0.4417	0.0971	0.051*	
O1B	0.24453 (15)	0.57634 (9)	0.41257 (8)	0.0228 (2)	
O2B	0.17672 (15)	0.39175 (8)	0.45092 (8)	0.0239 (2)	
O3B	0.08596 (16)	0.31742 (9)	0.32564 (9)	0.0257 (3)	
C1B	0.10480 (19)	0.42674 (12)	0.26094 (11)	0.0192 (3)	
C2B	0.0707 (2)	0.44786 (13)	0.16487 (12)	0.0223 (3)	
H2BA	0.0358	0.3880	0.1471	0.027*	
C3B	0.0891 (2)	0.55775 (13)	0.09701 (11)	0.0229 (3)	
H3BA	0.0664	0.5716	0.0333	0.027*	
C4B	0.1412 (2)	0.64860 (13)	0.12204 (11)	0.0234 (3)	
H4BA	0.1538	0.7225	0.0755	0.028*	
C5B	0.1739 (2)	0.62728 (13)	0.21704 (11)	0.0212 (3)	
H5BA	0.2080	0.6878	0.2340	0.025*	
C6B	0.15685 (19)	0.51694 (12)	0.28802 (11)	0.0174 (3)	
C7B	0.19489 (19)	0.49451 (12)	0.39042 (11)	0.0183 (3)	
N1B	0.26797 (18)	−0.00016 (10)	0.30554 (9)	0.0190 (3)	
N2B	0.34027 (19)	−0.18896 (11)	0.33877 (10)	0.0242 (3)	
C8B	0.0346 (2)	−0.14199 (13)	0.40050 (11)	0.0202 (3)	
H8BA	−0.0036	−0.2186	0.4305	0.024*	
C9B	0.2159 (2)	−0.11240 (12)	0.34789 (11)	0.0188 (3)	
C10B	0.1505 (2)	0.08329 (12)	0.31225 (11)	0.0203 (3)	
H10B	0.1904	0.1595	0.2817	0.024*	
C11B	−0.0243 (2)	0.05709 (13)	0.36292 (11)	0.0218 (3)	
H11B	−0.1028	0.1148	0.3680	0.026*	
C12B	−0.0857 (2)	−0.05856 (13)	0.40769 (10)	0.0207 (3)	
C13B	−0.2817 (2)	−0.08836 (15)	0.46052 (12)	0.0277 (4)	
H13D	−0.2952	−0.1695	0.4954	0.042*	
H13E	−0.3683	−0.0649	0.4138	0.042*	
H13F	−0.3067	−0.0497	0.5061	0.042*	
H1A3	0.743 (3)	0.1794 (19)	0.1574 (17)	0.061 (7)*	
H1NA	0.719 (3)	0.4528 (17)	0.4688 (16)	0.049 (6)*	
H2NA	0.748 (3)	0.7276 (17)	0.3090 (14)	0.035 (5)*	
H1B3	0.111 (3)	0.327 (2)	0.3833 (18)	0.063 (7)*	
H3NA	0.769 (3)	0.6423 (17)	0.4187 (16)	0.044 (6)*	
H1NB	0.390 (3)	0.0191 (17)	0.2677 (14)	0.046 (6)*	
H2NB	0.457 (3)	−0.1645 (16)	0.2974 (14)	0.039 (5)*	
H3NB	0.302 (2)	−0.2656 (16)	0.3608 (13)	0.029 (5)*	

### Atomic displacement parameters (Å^2^)

**Table:**

	*U*^11^	*U*^22^	*U*^33^	*U*^12^	*U*^13^	*U*^23^
O1A	0.0262 (6)	0.0192 (5)	0.0306 (6)	−0.0005 (4)	−0.0029 (5)	−0.0047 (5)
O2A	0.0183 (5)	0.0199 (5)	0.0321 (6)	0.0006 (4)	−0.0010 (4)	−0.0071 (5)
O3A	0.0233 (6)	0.0201 (5)	0.0297 (6)	−0.0014 (4)	0.0001 (5)	−0.0061 (5)
C1A	0.0193 (7)	0.0240 (7)	0.0168 (7)	0.0010 (6)	−0.0036 (6)	−0.0057 (6)
C2A	0.0231 (8)	0.0311 (9)	0.0268 (9)	−0.0053 (7)	0.0022 (6)	−0.0052 (7)
C3A	0.0234 (8)	0.0492 (11)	0.0310 (10)	−0.0004 (8)	0.0051 (7)	−0.0104 (8)
C4A	0.0281 (9)	0.0431 (10)	0.0323 (10)	0.0099 (8)	0.0014 (7)	−0.0145 (8)
C5A	0.0272 (8)	0.0280 (8)	0.0239 (8)	0.0064 (6)	−0.0042 (6)	−0.0099 (7)
C6A	0.0192 (7)	0.0234 (7)	0.0162 (7)	0.0026 (6)	−0.0040 (5)	−0.0062 (6)
C7A	0.0208 (7)	0.0206 (7)	0.0176 (7)	0.0015 (6)	−0.0063 (6)	−0.0046 (6)
N1A	0.0196 (6)	0.0233 (6)	0.0208 (7)	0.0000 (5)	−0.0031 (5)	−0.0073 (5)
N2A	0.0277 (7)	0.0210 (7)	0.0242 (7)	0.0021 (5)	−0.0069 (6)	−0.0057 (6)
C8A	0.0165 (7)	0.0309 (8)	0.0208 (8)	0.0033 (6)	−0.0028 (6)	−0.0052 (6)
C9A	0.0137 (6)	0.0239 (7)	0.0227 (8)	0.0024 (5)	−0.0021 (6)	−0.0067 (6)
C10A	0.0216 (7)	0.0223 (7)	0.0279 (8)	−0.0023 (6)	−0.0024 (6)	−0.0079 (6)
C11A	0.0218 (8)	0.0332 (9)	0.0330 (9)	−0.0034 (7)	−0.0017 (7)	−0.0171 (7)
C12A	0.0136 (7)	0.0395 (9)	0.0255 (8)	0.0008 (6)	−0.0022 (6)	−0.0138 (7)
C13A	0.0239 (8)	0.0534 (11)	0.0275 (9)	−0.0020 (8)	−0.0040 (7)	−0.0185 (8)
O1B	0.0295 (6)	0.0175 (5)	0.0216 (6)	−0.0026 (4)	−0.0058 (4)	−0.0065 (4)
O2B	0.0298 (6)	0.0162 (5)	0.0225 (6)	−0.0027 (4)	−0.0036 (5)	−0.0030 (4)
O3B	0.0318 (6)	0.0153 (5)	0.0302 (6)	−0.0019 (4)	−0.0077 (5)	−0.0070 (5)
C1B	0.0150 (6)	0.0170 (7)	0.0252 (8)	0.0019 (5)	−0.0024 (6)	−0.0073 (6)
C2B	0.0196 (7)	0.0245 (7)	0.0280 (8)	0.0012 (6)	−0.0054 (6)	−0.0149 (7)
C3B	0.0193 (7)	0.0297 (8)	0.0222 (8)	0.0031 (6)	−0.0055 (6)	−0.0117 (7)
C4B	0.0238 (8)	0.0223 (7)	0.0218 (8)	0.0008 (6)	−0.0036 (6)	−0.0053 (6)
C5B	0.0226 (7)	0.0194 (7)	0.0225 (8)	−0.0010 (6)	−0.0032 (6)	−0.0087 (6)
C6B	0.0132 (6)	0.0180 (7)	0.0207 (7)	0.0000 (5)	−0.0024 (5)	−0.0067 (6)
C7B	0.0152 (7)	0.0174 (7)	0.0209 (7)	0.0010 (5)	−0.0016 (5)	−0.0058 (6)
N1B	0.0184 (6)	0.0159 (6)	0.0217 (6)	−0.0014 (5)	−0.0032 (5)	−0.0053 (5)
N2B	0.0221 (7)	0.0158 (6)	0.0330 (8)	−0.0004 (5)	−0.0030 (6)	−0.0071 (6)
C8B	0.0214 (7)	0.0181 (7)	0.0188 (7)	−0.0048 (6)	−0.0033 (6)	−0.0034 (6)
C9B	0.0216 (7)	0.0162 (7)	0.0185 (7)	−0.0001 (5)	−0.0066 (6)	−0.0048 (6)
C10B	0.0235 (7)	0.0160 (7)	0.0214 (7)	0.0010 (6)	−0.0067 (6)	−0.0056 (6)
C11B	0.0229 (8)	0.0229 (7)	0.0216 (8)	0.0035 (6)	−0.0066 (6)	−0.0093 (6)
C12B	0.0208 (7)	0.0264 (8)	0.0148 (7)	−0.0008 (6)	−0.0056 (6)	−0.0061 (6)
C13B	0.0202 (8)	0.0356 (9)	0.0251 (8)	−0.0018 (7)	−0.0020 (6)	−0.0087 (7)

### Geometric parameters (Å, °)

**Table:**

O1A—C7A	1.2500 (18)	O1B—C7B	1.2580 (17)
O2A—C7A	1.2843 (16)	O2B—C7B	1.2722 (17)
O3A—C1A	1.3591 (17)	O3B—C1B	1.3549 (18)
O3A—H1A3	0.99 (2)	O3B—H1B3	0.94 (2)
C1A—C2A	1.396 (2)	C1B—C2B	1.402 (2)
C1A—C6A	1.402 (2)	C1B—C6B	1.404 (2)
C2A—C3A	1.380 (2)	C2B—C3B	1.376 (2)
C2A—H2AA	0.9300	C2B—H2BA	0.9300
C3A—C4A	1.385 (3)	C3B—C4B	1.393 (2)
C3A—H3AA	0.9300	C3B—H3BA	0.9300
C4A—C5A	1.384 (2)	C4B—C5B	1.383 (2)
C4A—H4AA	0.9300	C4B—H4BA	0.9300
C5A—C6A	1.3933 (19)	C5B—C6B	1.397 (2)
C5A—H5AA	0.9300	C5B—H5BA	0.9300
C6A—C7A	1.495 (2)	C6B—C7B	1.499 (2)
N1A—C10A	1.356 (2)	N1B—C9B	1.3550 (18)
N1A—C9A	1.3566 (19)	N1B—C10B	1.3584 (18)
N1A—H1NA	0.99 (2)	N1B—H1NB	0.96 (2)
N2A—C9A	1.329 (2)	N2B—C9B	1.3331 (18)
N2A—H2NA	0.90 (2)	N2B—H2NB	0.96 (2)
N2A—H3NA	0.93 (2)	N2B—H3NB	0.930 (19)
C8A—C12A	1.366 (2)	C8B—C12B	1.373 (2)
C8A—C9A	1.411 (2)	C8B—C9B	1.412 (2)
C8A—H8AA	0.9300	C8B—H8BA	0.9300
C10A—C11A	1.355 (2)	C10B—C11B	1.359 (2)
C10A—H10A	0.9300	C10B—H10B	0.9300
C11A—C12A	1.415 (2)	C11B—C12B	1.411 (2)
C11A—H11A	0.9300	C11B—H11B	0.9300
C12A—C13A	1.504 (2)	C12B—C13B	1.506 (2)
C13A—H13A	0.9600	C13B—H13D	0.9600
C13A—H13B	0.9600	C13B—H13E	0.9600
C13A—H13C	0.9600	C13B—H13F	0.9600
			
C1A—O3A—H1A3	103.3 (12)	C1B—O3B—H1B3	101.2 (14)
O3A—C1A—C2A	118.13 (14)	O3B—C1B—C2B	117.87 (13)
O3A—C1A—C6A	121.82 (13)	O3B—C1B—C6B	121.75 (13)
C2A—C1A—C6A	120.05 (14)	C2B—C1B—C6B	120.38 (13)
C3A—C2A—C1A	119.90 (16)	C3B—C2B—C1B	119.53 (13)
C3A—C2A—H2AA	120.0	C3B—C2B—H2BA	120.2
C1A—C2A—H2AA	120.0	C1B—C2B—H2BA	120.2
C2A—C3A—C4A	120.78 (16)	C2B—C3B—C4B	121.15 (14)
C2A—C3A—H3AA	119.6	C2B—C3B—H3BA	119.4
C4A—C3A—H3AA	119.6	C4B—C3B—H3BA	119.4
C5A—C4A—C3A	119.30 (15)	C5B—C4B—C3B	119.05 (14)
C5A—C4A—H4AA	120.4	C5B—C4B—H4BA	120.5
C3A—C4A—H4AA	120.4	C3B—C4B—H4BA	120.5
C4A—C5A—C6A	121.29 (16)	C4B—C5B—C6B	121.54 (14)
C4A—C5A—H5AA	119.4	C4B—C5B—H5BA	119.2
C6A—C5A—H5AA	119.4	C6B—C5B—H5BA	119.2
C5A—C6A—C1A	118.66 (14)	C5B—C6B—C1B	118.36 (13)
C5A—C6A—C7A	120.15 (14)	C5B—C6B—C7B	121.13 (13)
C1A—C6A—C7A	121.18 (12)	C1B—C6B—C7B	120.50 (13)
O1A—C7A—O2A	123.35 (14)	O1B—C7B—O2B	123.09 (14)
O1A—C7A—C6A	119.44 (12)	O1B—C7B—C6B	119.41 (13)
O2A—C7A—C6A	117.21 (13)	O2B—C7B—C6B	117.49 (12)
C10A—N1A—C9A	122.06 (14)	C9B—N1B—C10B	121.76 (13)
C10A—N1A—H1NA	118.1 (12)	C9B—N1B—H1NB	117.6 (12)
C9A—N1A—H1NA	119.8 (12)	C10B—N1B—H1NB	120.6 (12)
C9A—N2A—H2NA	119.0 (12)	C9B—N2B—H2NB	120.4 (11)
C9A—N2A—H3NA	118.2 (12)	C9B—N2B—H3NB	119.0 (11)
H2NA—N2A—H3NA	122.8 (17)	H2NB—N2B—H3NB	119.2 (16)
C12A—C8A—C9A	120.89 (15)	C12B—C8B—C9B	120.48 (13)
C12A—C8A—H8AA	119.6	C12B—C8B—H8BA	119.8
C9A—C8A—H8AA	119.6	C9B—C8B—H8BA	119.8
N2A—C9A—N1A	118.14 (14)	N2B—C9B—N1B	117.99 (13)
N2A—C9A—C8A	124.06 (15)	N2B—C9B—C8B	123.61 (13)
N1A—C9A—C8A	117.80 (14)	N1B—C9B—C8B	118.39 (13)
C11A—C10A—N1A	121.14 (15)	N1B—C10B—C11B	121.03 (14)
C11A—C10A—H10A	119.4	N1B—C10B—H10B	119.5
N1A—C10A—H10A	119.4	C11B—C10B—H10B	119.5
C10A—C11A—C12A	119.10 (15)	C10B—C11B—C12B	119.38 (13)
C10A—C11A—H11A	120.4	C10B—C11B—H11B	120.3
C12A—C11A—H11A	120.4	C12B—C11B—H11B	120.3
C8A—C12A—C11A	119.01 (15)	C8B—C12B—C11B	118.95 (13)
C8A—C12A—C13A	121.66 (16)	C8B—C12B—C13B	121.20 (14)
C11A—C12A—C13A	119.33 (15)	C11B—C12B—C13B	119.84 (13)
C12A—C13A—H13A	109.5	C12B—C13B—H13D	109.5
C12A—C13A—H13B	109.5	C12B—C13B—H13E	109.5
H13A—C13A—H13B	109.5	H13D—C13B—H13E	109.5
C12A—C13A—H13C	109.5	C12B—C13B—H13F	109.5
H13A—C13A—H13C	109.5	H13D—C13B—H13F	109.5
H13B—C13A—H13C	109.5	H13E—C13B—H13F	109.5
			
O3A—C1A—C2A—C3A	178.90 (15)	O3B—C1B—C2B—C3B	−179.74 (13)
C6A—C1A—C2A—C3A	−0.7 (2)	C6B—C1B—C2B—C3B	0.2 (2)
C1A—C2A—C3A—C4A	0.5 (3)	C1B—C2B—C3B—C4B	0.0 (2)
C2A—C3A—C4A—C5A	0.3 (3)	C2B—C3B—C4B—C5B	−0.3 (2)
C3A—C4A—C5A—C6A	−0.9 (3)	C3B—C4B—C5B—C6B	0.4 (2)
C4A—C5A—C6A—C1A	0.6 (2)	C4B—C5B—C6B—C1B	−0.1 (2)
C4A—C5A—C6A—C7A	−178.47 (15)	C4B—C5B—C6B—C7B	179.23 (13)
O3A—C1A—C6A—C5A	−179.39 (14)	O3B—C1B—C6B—C5B	179.82 (13)
C2A—C1A—C6A—C5A	0.2 (2)	C2B—C1B—C6B—C5B	−0.1 (2)
O3A—C1A—C6A—C7A	−0.4 (2)	O3B—C1B—C6B—C7B	0.4 (2)
C2A—C1A—C6A—C7A	179.26 (14)	C2B—C1B—C6B—C7B	−179.52 (12)
C5A—C6A—C7A—O1A	−1.0 (2)	C5B—C6B—C7B—O1B	−0.6 (2)
C1A—C6A—C7A—O1A	−179.99 (14)	C1B—C6B—C7B—O1B	178.75 (13)
C5A—C6A—C7A—O2A	178.56 (14)	C5B—C6B—C7B—O2B	−179.89 (13)
C1A—C6A—C7A—O2A	−0.5 (2)	C1B—C6B—C7B—O2B	−0.52 (19)
C10A—N1A—C9A—N2A	179.75 (14)	C10B—N1B—C9B—N2B	−179.26 (13)
C10A—N1A—C9A—C8A	−0.5 (2)	C10B—N1B—C9B—C8B	−0.3 (2)
C12A—C8A—C9A—N2A	179.56 (14)	C12B—C8B—C9B—N2B	179.36 (14)
C12A—C8A—C9A—N1A	−0.2 (2)	C12B—C8B—C9B—N1B	0.4 (2)
C9A—N1A—C10A—C11A	0.8 (2)	C9B—N1B—C10B—C11B	0.7 (2)
N1A—C10A—C11A—C12A	−0.5 (2)	N1B—C10B—C11B—C12B	−1.2 (2)
C9A—C8A—C12A—C11A	0.5 (2)	C9B—C8B—C12B—C11B	−0.9 (2)
C9A—C8A—C12A—C13A	−178.45 (14)	C9B—C8B—C12B—C13B	177.87 (14)
C10A—C11A—C12A—C8A	−0.2 (2)	C10B—C11B—C12B—C8B	1.3 (2)
C10A—C11A—C12A—C13A	178.81 (14)	C10B—C11B—C12B—C13B	−177.53 (14)

### Hydrogen-bond geometry (Å, °)

**Table:**

*D*—H···*A*	*D*—H	H···*A*	*D*···*A*	*D*—H···*A*
O3A—H1A3···O2A	0.99 (2)	1.61 (2)	2.5310 (16)	154 (2)
N1A—H1NA···O1B^i^	0.99 (2)	1.71 (2)	2.6965 (17)	174 (2)
N2A—H2NA···O1A^ii^	0.90 (2)	1.99 (2)	2.8645 (19)	164 (2)
O3B—H1B3···O2B	0.94 (3)	1.62 (3)	2.5179 (16)	158 (2)
N2A—H3NA···O2B^i^	0.94 (2)	1.91 (2)	2.8468 (18)	178 (2)
N1B—H1NB···O2A	0.96 (2)	1.76 (2)	2.7186 (17)	172.7 (17)
N2B—H2NB···O1A	0.96 (2)	1.84 (2)	2.7976 (18)	177.0 (16)
N2B—H3NB···O1B^iii^	0.93 (2)	1.88 (2)	2.8097 (19)	174.3 (13)
C8B—H8BA···O2B^iv^	0.93	2.47	3.357 (2)	159
C10B—H10B···O3B	0.93	2.38	3.039 (2)	128

Symmetry codes: (i) −*x*+1, −*y*+1, −*z*+1; (ii) *x*, *y*+1, *z*; (iii) *x*, *y*−1, *z*; (iv) −*x*, −*y*, −*z*+1.
